# Evaluating the effectiveness of intensive versus non‐intensive image interpretation education for radiographers: a randomised controlled trial

**DOI:** 10.1002/jmrs.314

**Published:** 2018-11-09

**Authors:** Michael J. Neep, Tom Steffens, Patrick Eastgate, Steven M. McPhail

**Affiliations:** ^1^ Department of Medical Imaging Logan Hospital Meadowbrook Queensland Australia; ^2^ Centre for Functioning and Health Research Metro South Health Brisbane Australia; ^3^ School of Public Health and Social Work Queensland University of Technology Kelvin Grove Brisbane Australia; ^4^ Institute of Health and Biomedical Innovation Queensland University of Technology Kelvin Grove, Brisbane Australia; ^5^ Department of Medical Imaging Princess Alexandra Hospital Brisbane Australia; ^6^ Department of Medical Imaging Sunshine Coast University Hospital Birtinya Australia

**Keywords:** Education, image interpretation, radiographers, randomised control trial

## Abstract

**Introduction:**

The purpose of this randomised controlled trial was to compare the effectiveness of intensive and non‐intensive formats of delivery of image interpretation education for radiographers.

**Methods:**

A multi‐centre, stratified (by years of experience) two group parallel arm, single blind, randomised controlled trial was conducted. Participants (*n* = 48) were allocated to one of two groups to receive image interpretation education: (1) intensive format (13.5 h over two consecutive days) (2) non‐intensive (sequential 90‐min tutorials delivered 1 week apart). Participants undertook x‐ray interpretation tests before education, at 1‐week post‐education completion and at 12‐week post‐education completion.

**Results:**

Image interpretation performance was not significantly different between groups at baseline. A generalised linear model indicated that participants who received intensive education format improved image interpretation performance by a greater margin than the group that received non‐intensive education at 1‐week (*P* = 0.002) and 12‐week (*P* < 0.001) follow‐up assessments.

**Conclusions:**

Although both formats of education delivery may be beneficial, the findings of this study have indicated that the intensive format of delivery was more effective at improving radiographers’ ability to interpret trauma radiographs in the weeks after completion of the image interpretation program.

## Introduction

Failure to identify fractures is the most common diagnostic error in emergency departments.^1^, ^2^ Radiographer commenting or Preliminary Image Evaluation (PIE)^3^ has been suggested as a potential mechanism for reducing these errors. A PIE is a brief written description provided by the performing radiographer, which describes findings of a radiographic examination at the time of image acquisition.^4^, ^5^, ^6^, ^7^, ^8^ The benefits arising from PIE are likely to be proportional to radiographers’ ability to detect and describe abnormalities on trauma radiographs. Consequently, training that improves radiographers’ interpretive skills is imperative. No prior study has compared delivery of different formats of image interpretation education for radiographers.

The purpose of this RCT was to compare the effectiveness of the same image interpretation education program delivered over a 2‐day period (intensive format) versus a series of shorter regular workshops (non‐intensive format). The primary intended effect of this education program was to enhance radiographers’ ability to detect and describe potential abnormalities on trauma radiographs. Secondary aims included examining participants’ ratings of their confidence, perceived image interpretation accuracy and opinions of the quality of education received.

## Methods

### Study design

**Figure 1:**
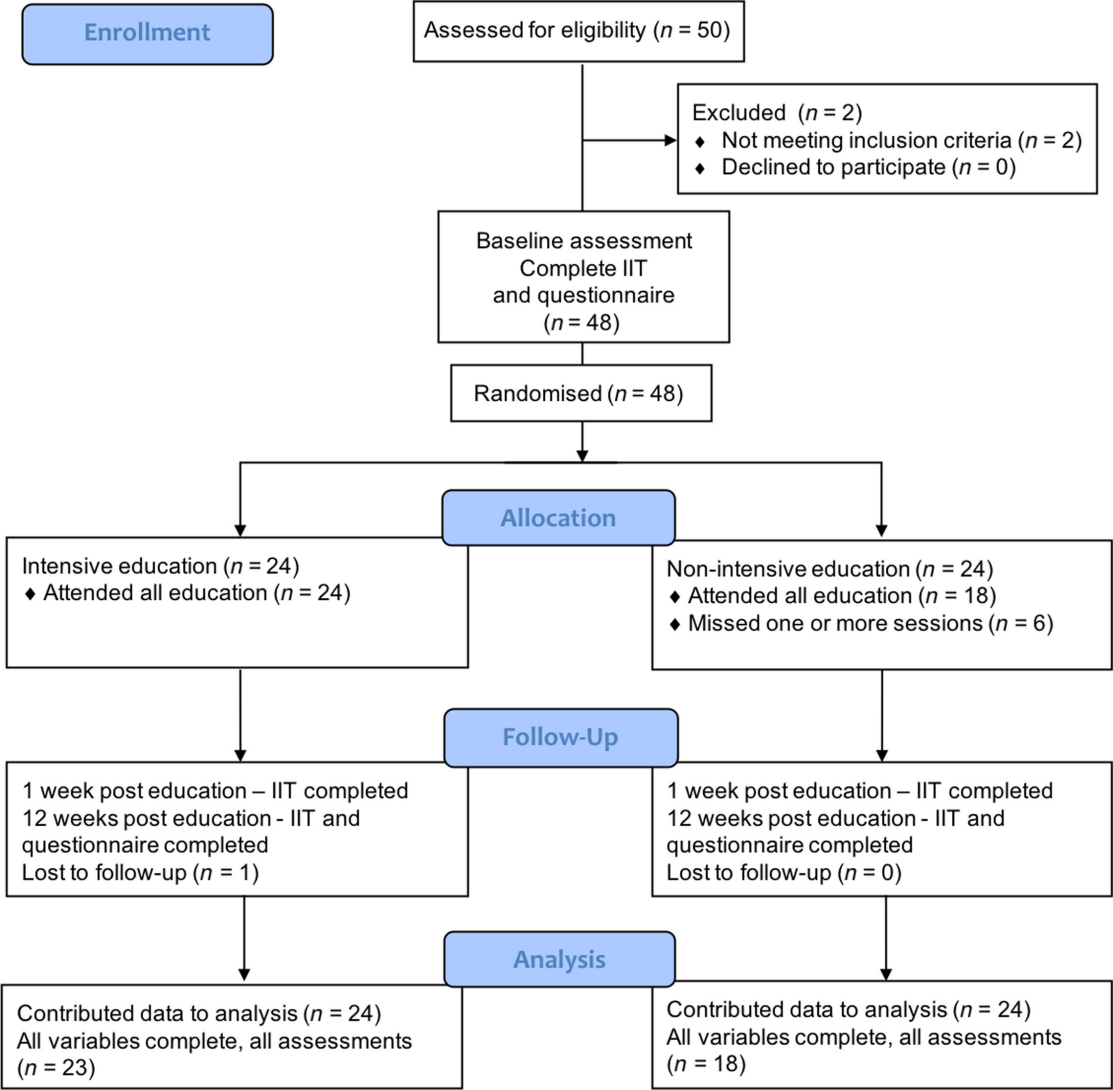
CONSORT flow diagram: Study design – randomised trial.

Details of the trial protocol have been previously described.^9^ In summary, this was a multi‐centre, stratified (by years of experience) two group parallel arm, single blind, (assessor blinded) randomised controlled trial (Fig. 1). Participants were allocated to one of two groups: (1) intensive format or (2) non‐intensive format in a 1:1 ratio. Participants completed assessments before education, at 1‐week post‐intervention completion and at 12‐week post‐intervention completion. The study period opened in September 2012, final recruitment closed in July 2017 and the last follow‐up assessments finalised in January 2018.

### Ethical considerations

Participants provided written informed consent with freedom to withdraw at any time. The study was approved by the Metro South Health (HREC/11/QPAH/172) and Queensland University of Technology (1200000061) ethics committees and prospectively registered (ACTRN12612000210875).

### Setting and participant recruitment

Radiographers were recruited from three hospitals with dedicated emergency departments in south east Queensland, Australia. Radiographers were eligible for inclusion if they were employed in an emergency‐imaging department and were agreeable to undertake either intensive or non‐intensive image interpretation training. Eligible radiographers were invited to participate via email. Radiographers were excluded if they had previously completed formal education in image interpretation (e.g. a masters degree that included image interpretation coursework), were not available to attend the proposed education at the scheduled times or were currently completing their graduate year (intern year).

### Sample size

The priori sample size calculation estimated that a target of 48 participants should be recruited.^9^ A sample size of 24 participants per group provided greater than 80% power to detect a 4‐point difference between groups in the primary outcome (IIT score) at a significance level of 0.05%, assuming a standard deviation of 4.5 and dropout rate <15%.^9^

### Randomisation

Participants were stratified by years of clinical experience (1–2; 3–5; 6–12; 13+ years) to minimise the risk of experience imbalance between groups. A computer‐generated, permuted block random number schedule was developed by a researcher (SMM) not involved in recruitment or assessments. Concealment of allocation occurred using opaque, consecutively numbered envelopes stored in a locked filing cabinet. One envelope was provided to site investigators (TS and MN) for each participant in order of recruitment at completion of their baseline assessment and opened to reveal group allocation. Each participant was allocated to either the intensive or non‐intensive format of education.

### Intervention

The education program was delivered in two formats: One group received intensive education (2‐day intensive format of delivery) and the other group received non‐intensive (sequential 90‐min tutorials delivered once per week).

Both formats contained identical educational content, for the same duration (13.5 h). The education program covered interpretation of appendicular and axial skeletal trauma and based on a successful program described previously.^9^ The program was divided into nine 90‐min workshops (Table 1). Key areas covered included the use of an original search strategy, how to structure the description of findings, common injuries, normal variants and frequently missed abnormalities. To standardise intervention delivery, the same two instructors, both of whom had considerable experience in image interpretation and facilitation of training, delivered both formats. The facilitators were neither aware of the contents of the assessments nor involved with marking the assessments. Furthermore, because the total educational content delivery time was standardised, this promoted equivalent in‐class learning experience for both groups regarding the facilitator time attributed to each component of the education.

**Table 1:** Education intervention content outline

Workshop	Subject
1	General principles and strategy for interpretation of skeletal trauma
2	Hand, wrist and forearm
3	Face including mandible
4	Foot, ankle and tibia/fibula
5	Knee and distal femur
6	Pelvis and hips
7	Shoulder and humerus
8	Spine
9	Review of all content

### Outcome measures

#### Primary outcome measure

The image interpretation test (IIT) assessment score was used to determine which format of delivery resulted in greater improvement and maintenance of image interpretation ability. The development of this instrument, as well as evidence supporting its validity and reliability for examining radiographers’ image interpretation ability have been described previously.^10^ The IIT required participants examining a test bank of radiographic examinations (presented in random order) to identify abnormalities (and provide a descriptive comment when an abnormality was observed). The IIT contained 60 cases presented in DICOM format (Digital Imaging and Communications in Medicine). It included various appendicular and axial adult skeletal radiographs with a distribution of anatomical regions representative of a typical case mix from an adult hospital emergency department. The proportion of normal and abnormal cases in the IIT was also consistent with typical clinical practice in adult hospital emergency departments.^10^

The IIT was completed prior to education commencement, 1‐week post‐education completion and at 12‐week post‐education completion. Before each assessment, radiographers were provided with a guideline for classification of each radiographic examination consistent with prior research in the field.^11^ This guideline indicated that a normal finding should include anatomical variants, non‐traumatic pathology, old fractures and evidence of previous surgery, unless specifically related to the presentation. In contrast, an abnormal finding would include joint effusions, fractures, dislocations, subluxations and soft tissue swelling. Prior to interpreting and commenting on radiographs in the IIT, each participant received instruction on how to use the image review software (Codonics Clarity Viewer version 6.1, Middleburg Heights, Ohio, USA). This software provided the participant with functionality consistent with the clinical setting where they could adjust image contrast/density, zoom, pan and invert an image. To simulate the clinical setting and typical workflow, the assessment took place in a semi‐darkened room with a time restriction. Ninety minutes was assigned to complete the assessment, consistent with the prior validation of the IIT.^10^ Participants were permitted to complete the IIT cases in any order which included whether or not to interpret or skip past a particular case.

Two radiographers with postgraduate qualifications in image interpretation served as a panel of independent raters to score each case completed by participants against the reference standard. Both raters were blinded to group allocation. The scoring criteria (Table 2) and reference standard were consistent with the prior validation of the IIT.^10^ The raters were trained to use the scoring criteria by the site investigator (MN) and were provided with a marking guide and worksheet to ensure a consistent framework for marking. By using the scoring criteria each case in the IIT was given a numerical value with a maximum total score of 3 and minimum of 0 (the theoretical maximum score for the 60 IIT items was 180). A third independent rater was available to mediate any disagreements between the two primary raters. In addition, because the IIT is a timed test, the number of items not attempted was recorded at each assessment.

**Table 2:** Scoring criteria for each examination in the image interpretation test

Criteria	Score
For radiographic cases with a traumatic abnormality	
Abnormality not detected	0
Abnormality detected, but not described correctly	1
Abnormality detected, description incomplete (but not incorrect)	2
Abnormality detected and correctly described in entirety	3
For radiographic cases with no traumatic abnormality	
False abnormality reported or described	0
Correct report of absence of any traumatic abnormality	3

#### Secondary outcome measures

In addition to providing a description of the pathology (perceived to be) present, the participants provided a ‘confidence rating’ on a 5‐point Likert scale (normal, probably normal, possibly normal, probably abnormal and abnormal). Confidence ratings were scored for each case from 5 to 1 for normal cases and 1 to 5 for abnormal cases across the respective Likert response options. This scoring approach ensured that confident but incorrect ratings were awarded the lowest score (1), while definitive correct ratings were awarded the highest score (5) for confidence ratings on each IIT case. These ratings were recorded at baseline and at both follow‐up assessments.

Participants also completed a questionnaire on two occasions. The first was completed following the baseline IIT (but before randomisation) and the second was completed after the 12‐week assessment. The first questionnaire included a series of ratings using 11‐point Likert scales. Specifically, participants were asked to rate (self‐perception) their confidence in detecting and describing abnormalities, confidence to participate in radiographer commenting and accuracy in detecting and describing abnormalities of the appendicular and axial skeleton. In each case 0 represented ‘not at all (confident or accurate)’ and 10 represented ‘very (confident or accurate)’. The same ratings were completed after the 12‐week post‐education assessment. In addition, at the 12‐week post‐education assessment, participants were asked to rate statements about the volume, complexity and intensity of the education they received (0 represented strongly disagree and 10 represented strongly agree).

### Statistical analysis

Outcome measures were compared between groups at baseline using unpaired between‐group comparisons. Mixed effects generalised linear models (Poisson family for total counts of item scores, Gaussian family for numeric questionnaire ratings) were used to examine changes occurring at 1‐ and 12‐week post‐intervention assessments in comparison to baseline, as well as whether there was difference in the amount of change over time between those who received the intensive versus non‐intensive format (group by time interaction). From these analyses, it was possible to determine which method of education delivery (if any) had a greater impact on improving radiographers’ (a) ability to detect and describe abnormalities on trauma radiographs on the IIT (b) self‐rated (perceived) interpretation accuracy and (c) confidence in image interpretation, with the opportunity to adjust for baseline confounders in each of these three models, if indicated. There was no evidence of differences between groups at baseline and findings were entirely consistent whether or not potential confounders were included in the generalised linear models. Therefore, results from the unadjusted models have been presented. In addition, box plots were prepared to visualise the performance of participants in each group at each of the three assessments.

The aforementioned analyses were conducted following the intention‐to‐treat principle. Of the 144 assessment points (48 participants × 3 assessments), 13 (9%) assessments contained missing data. All missing data were from participants who dropped out prior to completing all follow‐up assessments. Wilcoxon rank‐sum tests confirmed there were no significant differences at baseline across the primary and secondary outcomes between participants who did and did not dropout. All available data from the 131 (91%) completed assessments were used in the generalised linear mixed models for primary analyses. To examine the potential impact of any missing data, sensitivity analyses were conducted using last value carried forward as well as multiple imputation using chained equations (M = 20).^12^ However, findings were consistent regardless of how missing data were treated, therefore the primary analyses with complete assessments have been presented.

## Results

### Participant characteristics

Forty‐eight participants were recruited (24 participants allocated to each trial arm) and 42 (88%) completed the training (Fig. 1). Six participants dropped out of the non‐intensive program prior to completion. No participants dropped out of the intensive program, although one participant from the intensive group was not available to complete all follow‐up assessments. At baseline, there were no between‐group differences in age, gender or years of clinical experience. The median (IQR) years of clinical experience were 4 (2–7) and 3 (2–6) for the intensive and non‐intensive arms respectively. The median (IQR) age was 27 (24–31) for the intensive group and 27 (25–34) for non‐intensive. There were 16 (67%) females in the intensive group and 14 (58%) in the non‐intensive group.

### Primary outcome – image interpretation test

**Figure 2:**
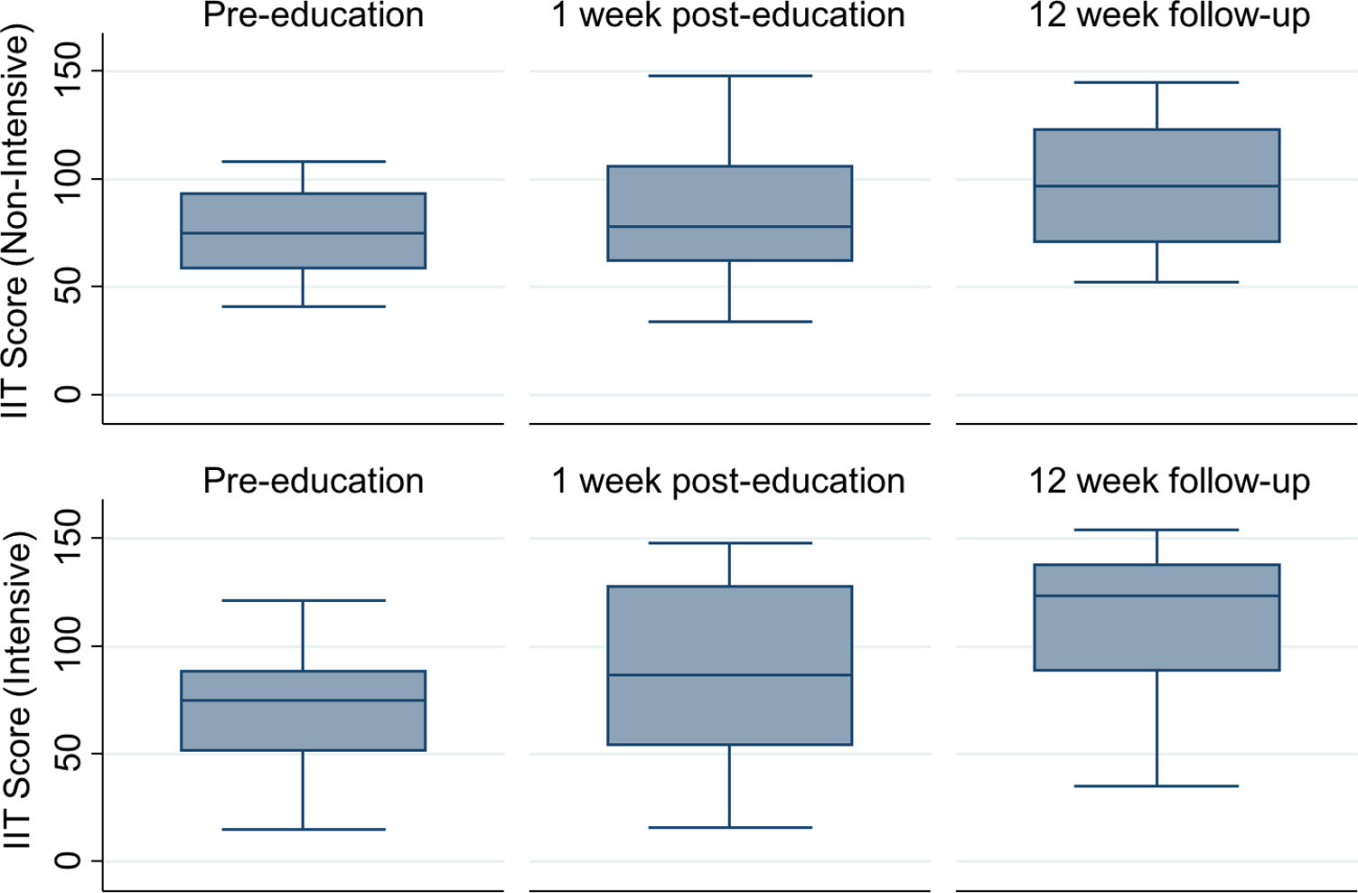
Box plots of image interpretation test scores (by group and assessment).

There was no significant difference in total IIT score at baseline between the intensive (median (IQR) = 75 (52–88)) and non‐intensive (median (IQR) = 75 (59–93)) formats. Both groups had higher 1‐week median (IQR) post‐education IIT scores (intensive: 87 (54–128); non‐intensive: 78 (63–106)) and 12‐week post‐education IIT scores (intensive: 124 (89–138); non‐intensive: 97 (71–123)) compared to baseline assessments. The IIT generalised linear mixed model indicated significant improvement across the entire sample (both groups) at 1‐week (coefficient (95% CI) = 0.20 (0.15–0.25), *P* < 0.001) and 12‐week post‐education assessment (coefficient (95% CI) = 0.41 (0.36–0.45), *P* < 0.001). The group by time interaction indicated that the intensive group improved by a greater margin at the 1‐week (coefficient (95% CI) = 0.11 (0.01–0.22), *P* = 0.03) and 12‐week (coefficient (95% CI) = 0.15 (0.05–0.24), *P* < 0.01) post‐intervention assessments than the non‐intensive group. This can also be seen in Figure 2 where the box plots indicated there was a greater propensity for improvement in IIT score among the intensive group.

The median (IQR) number of unattempted IIT cases at baseline assessment was comparable for both trial arms (intensive: 13 (0–24); non‐intensive: 14 (3–20)). A significant effect of time‐point from the linear mixed models (inclusive of both groups) indicated that the number of unattempted cases was higher than baseline at the 1‐week post‐intervention assessments (coefficient (95% CI) = 0.39 (0.28–0.50), *P* < 0.001), but lower than baseline at the 12‐week post‐assessments (coefficient (95% CI) = −0.14 (−0.26 to −0.02), *P* < 0.01). However, the median (IQR) of unattempted IIT cases for the intensive group was lower than the non‐intensive group at both the 1‐week (intensive: 17 (0–34); non‐intensive: 23 (12–28)) and 12‐week follow‐up assessments (intensive: 6 (0–14); non‐intensive: 14 (4–21)). The group by time interaction also indicated the intensive group had fewer unattempted IIT cases at both the 1‐week (coefficient (95% CI) = −0.20 (−0.40 to 0.02, *P* = 0.07), and 12‐week follow‐up assessments (coefficient (95% CI) = −0.46, (−0.71 to −0.21), *P* < 0.001).

### Secondary outcome – item classification confidence

The median (IQR) sum of confidence ratings for IIT cases at baseline was comparable for both trial arms (intensive: 184 (141–215); non‐intensive: 186 (163–216)). The significant effect of time‐point from the linear mixed models (inclusive of both groups) indicated that confidence was lower than baseline at 1‐week post‐intervention assessments (coefficient (95% CI) = −0.11, (−0.17 to −0.08), *P* < 0.001), but higher than baseline at 12‐week post‐intervention assessments (coefficient (95% CI) = 0.07, (0.03–0.10), *P* < 0.001). However, the median (IQR) confidence ratings for the intensive group were higher than the non‐intensive group at both the 1‐week (intensive: 168 (101–230); non‐intensive: 150 (128–202)) and 12‐week follow‐up assessments (intensive: 220 (178–237); non‐intensive: 188 (159–216)). The group by time interaction also confirmed the intensive group had higher confidence ratings from IIT cases than the non‐intensive group at both the 1‐week (coefficient (95% CI) = 0.11 (0.04–0.17), *P* < 0.001), and 12‐week follow‐up assessment (coefficient (95% CI) = 0.13, (0.06–0.19), *P* < 0.001).

Median (IQR) for each of the questionnaires’ confidence and accuracy numeric ratings are presented for each group in Table 3. The linear mixed models indicated that both groups improved at 12‐week follow‐up assessment when compared to baseline across all self‐perception questionnaire ratings (*P* value range <0.001 to 0.001). However, only the rating of ability to detect abnormalities of the axial skeleton had a significant (*P* < 0.05) group by time interaction that indicated a greater increase observed among the intensive group at the 12‐week assessment. For the remainder of questionnaire ratings neither group's self‐rated confidence improved by a greater margin than the other, at follow‐up assessment. Similarly, there were no significant between‐group differences in participants’ median (IQR) ratings of the volume (intensive: 8 (7–10); non‐intensive: 8 (8–10) or complexity (intensive: 9 (7–10); non‐intensive: 9 (8–10)) of educational content. For ratings of whether the education received was too intensive, both groups provided ratings at the low end of the scale indicating participants did not find education delivery to be too intense. However, the intensive group's median (IQR) rating trended towards being a little higher than the non‐intensive group (intensive: 3, (2–5); non‐intensive: 1, (1–3), coefficient (95% CI) = 1.36, (−0.16 to 2.87), *P* = 0.08).

**Table 3:** Radiographers’ perceived confidence and accuracy regarding image interpretation ability

	Intensive group		Non‐intensive group	
Topic	Baseline (*n* = 24)	12‐week follow‐up (*n* = 23)	Baseline (*n* = 24)	12‐week follow‐up (*n* = 18)
	Median (IQR)	Median (IQR)	Median (IQR)	Median (IQR)
Confidence				
Detecting traumatic abnormalities	6 (5–7)	8 (6–9)	6 (6–7)	8 (7–8)
Describing traumatic abnormalities	5 (3–6)	7 (5–8)	5 (4–5)	6 (5–8)
To participate in radiographer commenting	7 (5–8)	8 (7–9)	6 (6–7)	8 (7–8)
Accuracy				
Detecting appendicular traumatic abnormalities	7 (6–8)	8 (7–9)	7 (6–8)	8 (7–8)
Describing appendicular traumatic abnormalities	6 (5–6)	7 (6–8)	6 (4–6)	7 (5–8)
Detecting axial traumatic abnormalities	6 (4–7)	7 (6–8)	7 (5–7)	7 (6–7)
Describing axial traumatic abnormalities	5 (4–6)	6 (5–7)	5 (4–6)	6 (5–7)

0 represented ‘not at all (confident or accurate)’ and 10 represented ‘very (*confident or accurate)’.

There were no adverse events in this study.

## Discussion

This was the first RCT comparing the effectiveness of intensive and non‐intensive formats of delivery of image interpretation education for radiographers. Although both formats of delivery may be beneficial, the intensive format was more effective at improving radiographers’ ability to interpret trauma radiographs in the weeks after completing the image interpretation education program. Participants who completed the intensive format also reported better confidence in their image interpretations than the non‐intensive group. One of the key findings was that the non‐intensive intervention arm experienced a greater number of dropouts, which may reflect the challenges encountered by participants committing to a series of nine short workshops compared to a 2‐day intensive program. This higher dropout rate (12%) for the non‐intensive education delivery in comparison to the intensive (0%), suggests potential pragmatic advantages in addition to educational outcome advantages.

Several prior studies have explored the effect of image interpretation education on radiographers’ ability to interpret radiographs.^7^, ^13^, ^14^, ^15^, ^16^, ^17^ The results of prior studies were encouraging with each reporting beneficial effects. However, unlike the current study, these studies did not use an RCT design and there has been no previous comparison of different formats of education delivery. In the current study, the radiographers in both trial arms yielded higher test scores at 1‐ and 12‐week follow‐up assessments when compared to baseline scores. Two previous studies^14^, ^16^ employed an immediate assessment following education and a follow‐up assessment similar to the current study. Utilising a 42 case test bank of radiographs, the accuracy of radiographer interpretation decreased from 71.4% to 65.47% following 2 days of education.^14^ However, at 6–10 weeks after education, the accuracy had improved to be greater than pre‐education level (80.95% vs. 71.4%). A similar finding was demonstrated in study performed by McConnell et al.,^16^ which utilised a test bank of 102 appendicular radiographs. This study demonstrated a pre‐education radiographer accuracy of 82%, which decreased to 81.4% following the education and exhibited an improved accuracy of 86.8% 8–10 weeks post‐education. Although these studies found improvements in image interpretation ability at final assessment, they reported a reduction in the performance immediately after education. They postulated that subsequent increase in performance was possibly due to the direct effect of completing the course and the period of time (6–10 weeks) in which to reflect on the content included in the education. Two other studies^15^, ^17^ incorporated longer follow‐up assessments following education. Smith et al^15^ used a 25 case image bank to assess 16 radiographers’ ability to interpret axial and appendicular radiographs. They were assessed before an education program and 6 months after. The pre‐ and post‐education accuracy was not statistically significant (57.3% vs. 61.0%). Mackay^17^ assessed 133 radiographers’ ability to detect traumatic pathology following a 2‐day education program. Using a 30 case image bank, assessments were completed before education, immediately following education and at 6 months. The results demonstrated that radiographers’ sensitivity to detect pathology significantly improved between the pre‐ (78.9%) and immediate (88.2%) assessments. However, at the 6 months assessment it fell below the baseline sensitivity (76.5%). Interestingly, both studies found that the benefit of education had dissipated by 6 months after training. This suggests that radiographers need ongoing training to maintain their skills. This would be particularly pertinent in medical imaging departments where a PIE system has not been embedded. The authors of the current study propose that the increase in performance found at both 1‐ and 12‐week follow‐up assessments was likely due to the effectiveness of the education program and participants practising their acquired skills while working in the clinical setting between the end of the education program and the final assessment. It would be worthwhile to explore whether this enhanced performance is maintained over a longer period (e.g. beyond 6 months). It is interesting to note that the number of unattempted cases on the timed IIT increased at 1‐week assessment for both groups, indicating participants were not yet time‐efficient in applying their recently acquired image interpretation skills. However, the non‐intensive group attempted fewer cases than the intensive group during both 1‐ and 12‐week follow‐up assessments. Furthermore, the intensive group completed more cases at 12‐week assessment than at baseline assessment (and achieved a higher IIT score indicating greater accuracy). These findings support the conclusion that the intensive format of delivery was more effective than the non‐intensive format.

There is a paucity of literature that reports radiographers’ confidence in their ability to interpret radiographs. Coleman and Piper^18^ was one study that assessed radiographers’ accuracy and their confidence to interpret a 20 case image bank of appendicular radiographs. Their findings revealed a moderate positive correlation (*r* = 0.51) between radiographers’ mean confidence in their image interpretation ability and their actual test score accuracy (*P* = 0.02). To the authors’ knowledge, the current study was the first study that assessed radiographers’ image interpretation confidence before and after education. In the current study, the questionnaire findings indicated that radiographers’ image interpretation confidence improved at the 12‐week rating when compared with baseline; however, self‐reported confidence changes were similar across groups. Interestingly, this finding was in contrast to the confidence ratings of specific cases in the IIT, where scoring rewarded confidence in correct interpretation of each case and penalised confidence in incorrect interpretation. This finding differs from the Coleman and Piper^18^ study. The current study highlights the importance of including a quantitative measure of image interpretation performance, such as the IIT, when evaluating image interpretation education.

A study published in 2014^4^ identified that radiographers considered targeted image interpretation education to be desirable, regardless of intensity. Prior research in other fields has examined the merits of intensive and non‐intensive education formats.^19^, ^20^, ^21^ The results of the current study were consistent with these prior studies’ findings, which have indicated that intensive formats may lead to comparable or slightly more favourable learning outcomes than non‐intensive.^19^, ^20^, ^21^

Methodologically, this study exhibits several strengths. Within the field of radiographer PIE education, this study represents the largest sample size and a robust RCT research design. A further strength is the inclusion of participants from three centres, adding support to the likelihood that findings can be generalised. In this study, the use of a longer‐term follow‐up (12‐weeks post‐education) can be considered a strength, but further research investigating whether performance continues to improve, is maintained or diminishes beyond 12 weeks is likely to be worthwhile. Another strong aspect of the study was that the primary outcome measure had undergone testing which supported its validity and reliability among radiographers.^10^

There are several limitations in this study that should be considered. There remains disparity in the literature as to whether manufactured image tests (i.e., hand‐picked cases) are an accurate indicator of interpretive performance in comparison to image test banks that have been developed to represent clinical practice. A study by Hardy et al^22^ investigated radiographers’ image interpretation performance on manufactured image banks versus clinical practice image banks. The results indicated that the manufactured image banks that contained a higher abnormality prevalence may overestimate abnormality detection ability. Therefore, caution is required when extrapolating the results of the current study that employed an image bank that represented typical clinical practice versus a manufactured image bank. The possibility of recall bias is a potential limitation, as participants completed the same assessment during a relatively short time frame. To minimise this, two preventative measures were employed. Firstly, a minimum time of 5 weeks elapsed between any two assessments. Secondly, a computer‐generated randomisation sequence was used to present the 60 IIT cases random order, further limiting potential for case recall associated with sequential cases. A further limitation of this study is that the IIT only contained adult trauma radiographs and consequently performance on the IIT may not reflect radiographers’ abilities to interpret other types of radiographs, for example, paediatric or non‐trauma. Although validated, the unique scoring criteria utilised in this study can be considered a limitation. Last, it was interesting to note that the assumptions for the a priori sample size estimate did not match the distribution of primary outcome data from the trial. Nonetheless, because a significant between‐group difference on this measure was observed, Type II error did not occur.

In summary, findings suggest that the intensive format of delivery was more effective at improving radiographers’ ability to interpret trauma radiographs, although participants demonstrated improvement in image interpretation ability in both trial arms. These findings may be of great relevance to health care providers, emergency department and medical imaging department directors seeking to improve radiographers’ or any other health professionals’ image interpretation ability. Future research could explore whether image interpretation ability is maintained, improves or diminishes beyond the 12 weeks assessed in this study.

## Conflict of Interest

MN and PE are directors of Egg Cup Training. The other authors declare that they have no competing interests.
